# Leader neurons drive spontaneous and evoked activation patterns in cortical networks

**DOI:** 10.1186/1471-2202-14-S1-P64

**Published:** 2013-07-08

**Authors:** Valentina Pasquale, Sergio Martinoia, Michela Chiappalone

**Affiliations:** 1 Department of Neuroscience and Brain Technologies - NTECH, Istituto Italiano di Tecnologia, Via Morego 30, Genova 16163, Italy; 2 Department of Informatics, Bioengineering, Robotics, System Engineering (DIBRIS), University of Genova, Genova 16145, Italy

## 

Recent studies about the generation and propagation of coordinated activity in cultured neuronal networks reported the existence of privileged neurons that consistently fire earlier than others at the onset of synchronized bursting events (or network bursts, NB), which have been termed major burst leaders (MBL) [1]. At the same time, by stimulating the network from different channels one can obtain very different responses, not only in size and delay but also in the activation order of the responding neurons [2].

We electrically stimulated rat cortical networks cultured on micro-electrode arrays from different locations, either MBL or non-MBL. We evaluated the intensity and the delay of responses from either MBL or non-MBL and we also compared the spontaneous and the evoked activation patterns.

**Figure 1:**
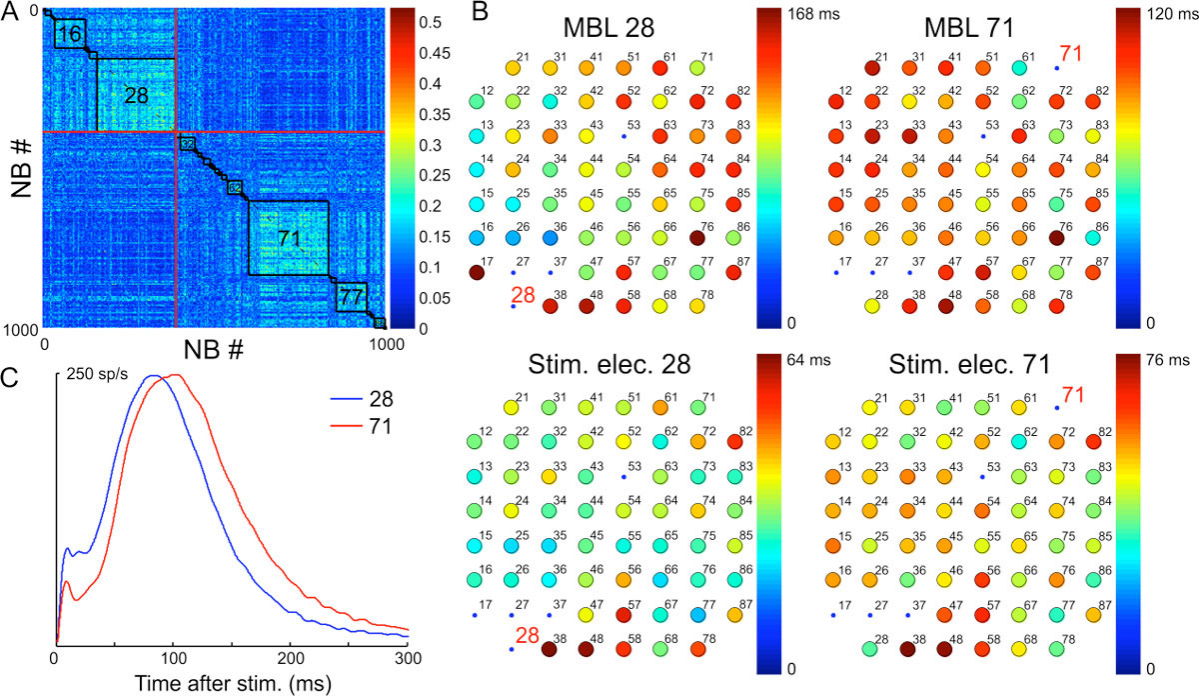
**A. Similarity matrix of activation order for 1000 spontaneous NB, ranked according to the MBL (indicated by numbers)**. Two distinct propagation patterns are visible. **B**. Color maps of average delays of followers with respect to either MBL spontaneous activation (upper panels) or external stimulation (lower panels). Spontaneous NB starting from 28 propagate rightward, whereas NB from 71 go leftward. NB evoked by stimulation of the same channels follow a similar pattern, although on a different timescale. **C**. Network average PSTH (for 28 and 71).

By comparing the responses obtained by stimulating either MBL or non-MBL, we found that the stimulation from MBL induces on average earlier responses. By comparing the responses of MBL and non-MBL, we found that MBL respond better and more rapidly to the stimulation coming from any other site in the network. Some networks showed different spontaneous propagation patterns within synchronized bursts depending on the identity of the corresponding MBL (Figure 1). In these cases the evoked propagation patterns correlate with the spontaneous ones and also depend on the spatial location of the stimulating site with respect to MBL.

To summarize, we demonstrated that MBLs do not only drive the propagation of coordinated spontaneous activations, but also play a special role in coordinating and driving the evoked bursts of activity.
